# Supplementary material for: The RAL Small G Proteins Are Clinically Relevant Targets in Triple Negative Breast Cancer
Source: Cancers (Basel). 2024 Aug 31;16(17):3043. doi: 10.3390/cancers16173043 (PMC11394424; doi:10.3390/cancers16173043)

# Full Western Blots

Cancers-2852472

# Figure 2A

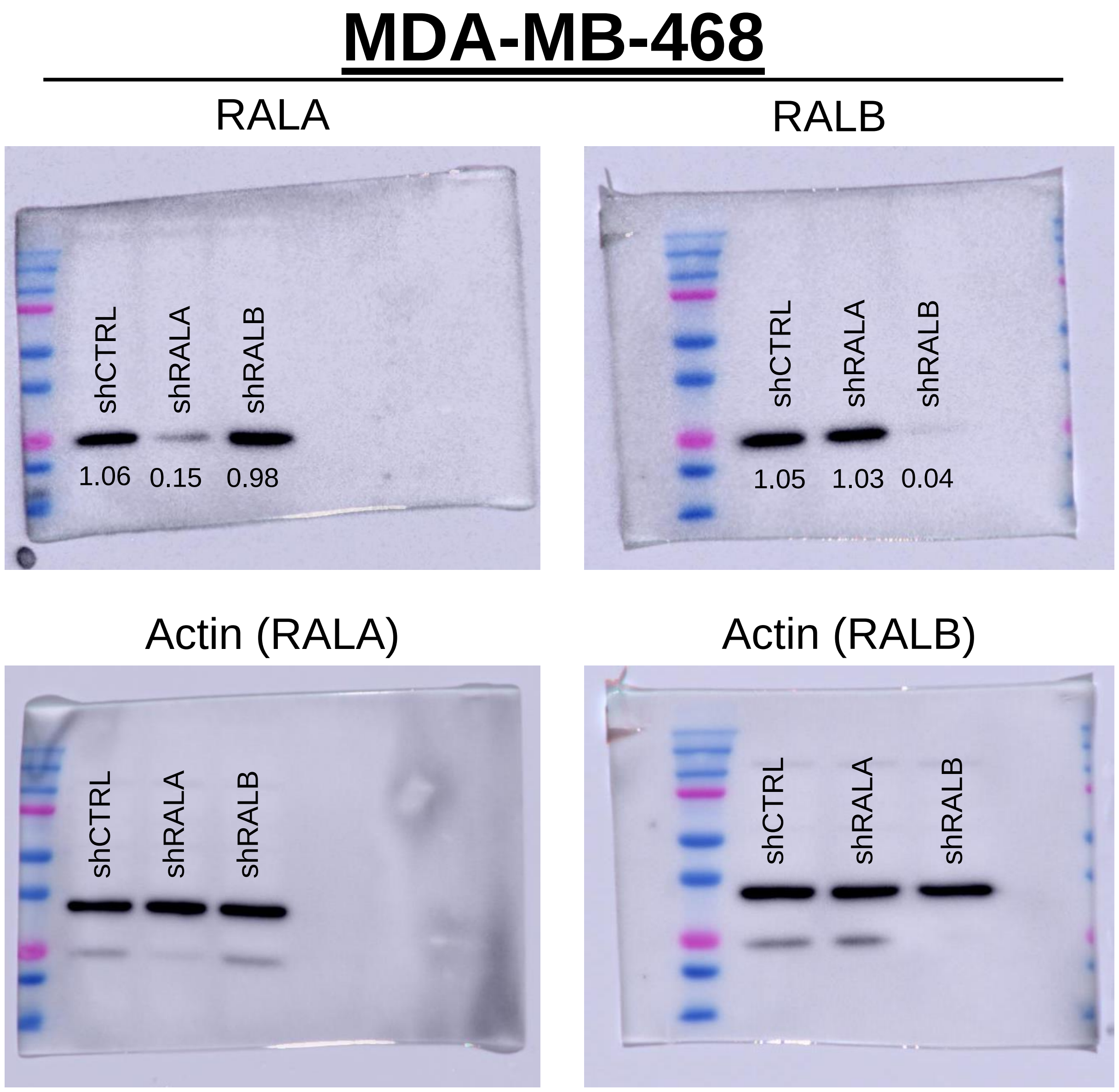
MDA-MB-468
RALA
RALB
shCTRL
shRALA
shRALB
1.06
0.15
0.98
1.05
1.03
0.04
Actin (RALA)
Actin (RALB)

Figure 3A

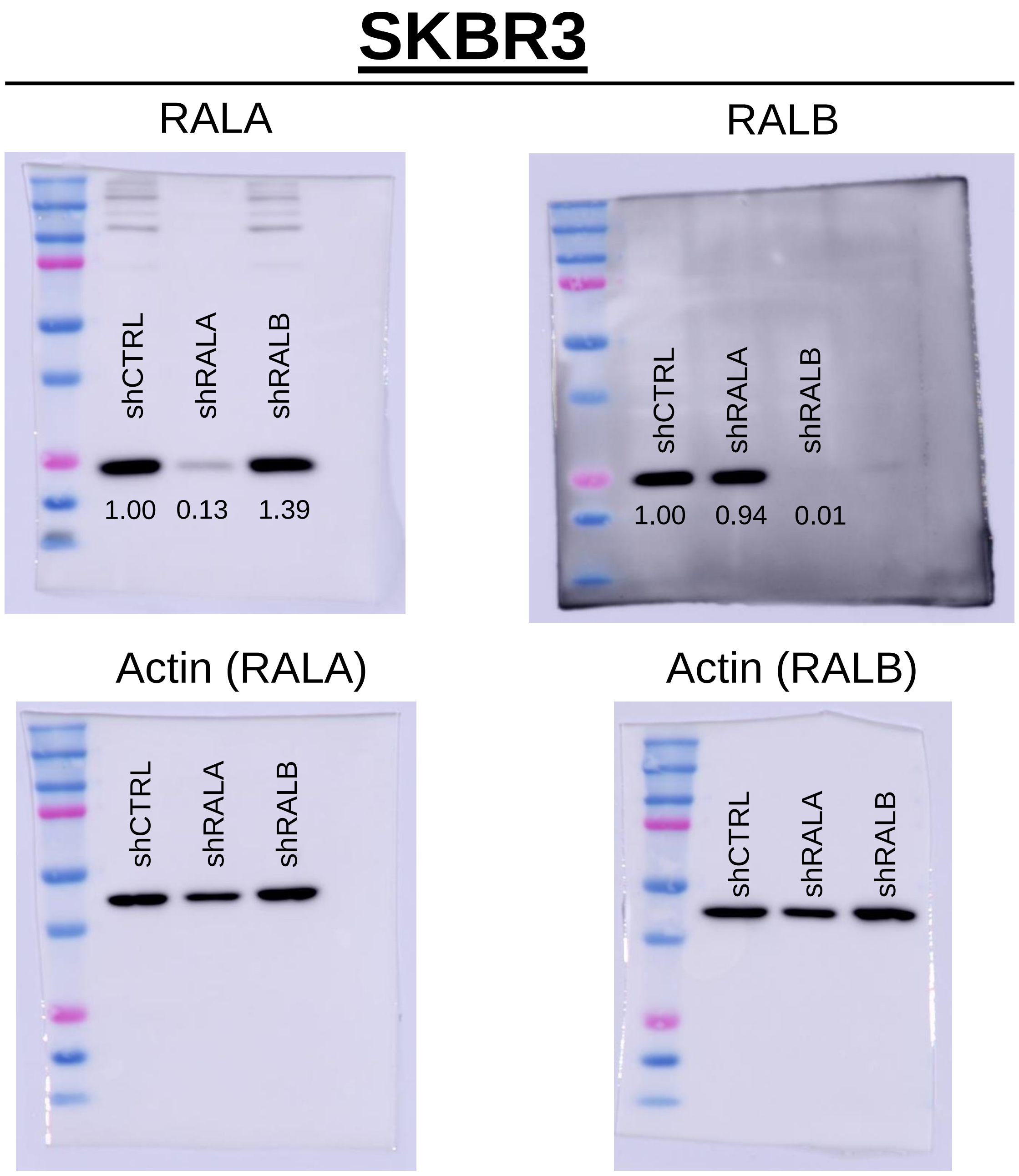

SKBR3
RALA
RALB
shCTRL
shRALA
shRALB
1.00
0.13
1.39
shCTRL
shRALA
shRALB
1.00
0.94
0.01
Actin (RALA)
Actin (RALB)
shCTRL
shRALA
shRALB
shCTRL
shRALA
shRALB

# Figure 7B

**Total RALA**

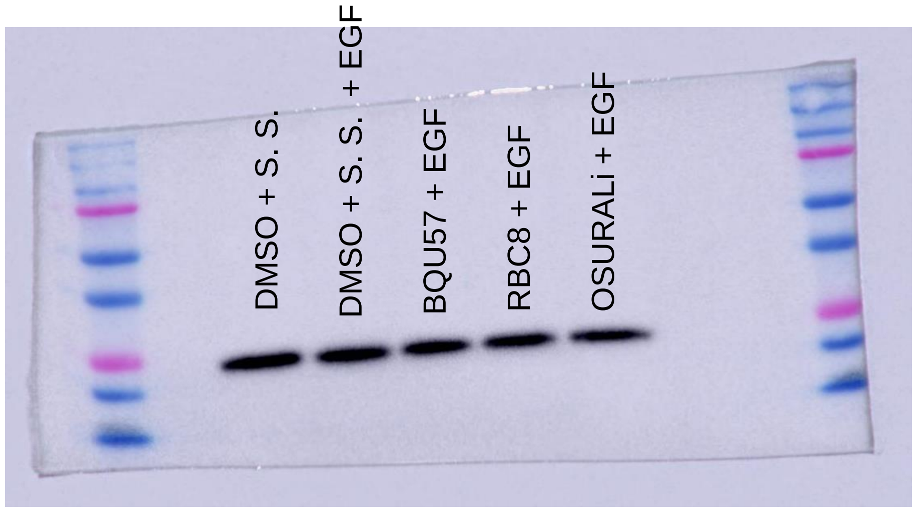


**Total RALB**

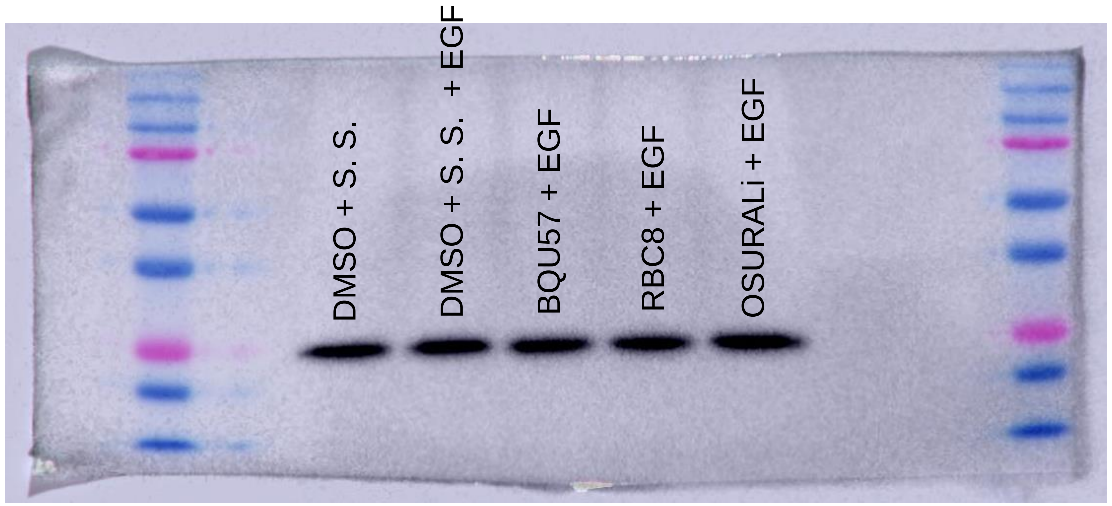


**GTP-RALA**

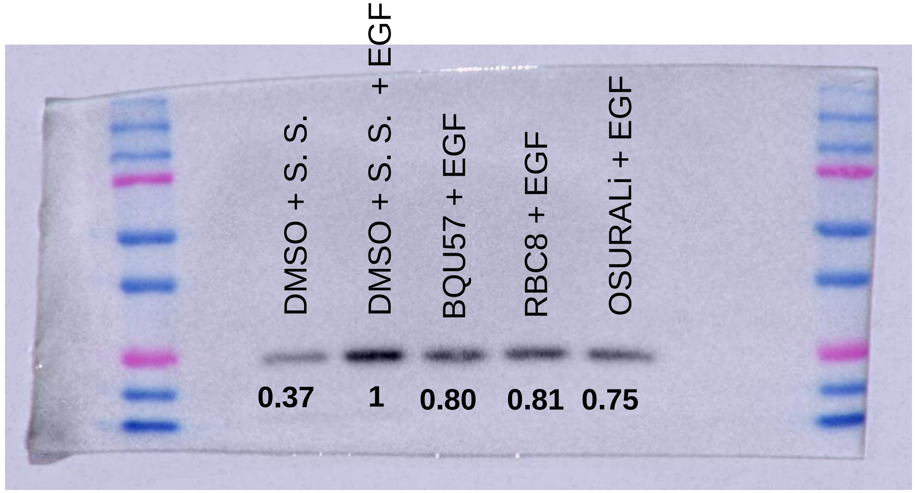


**GTP-RALB**

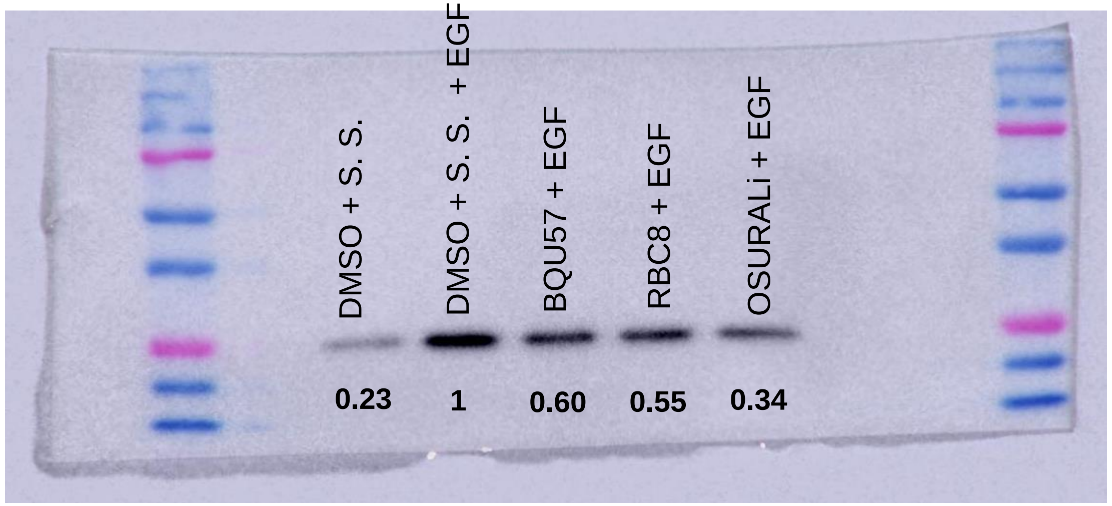

Supplementary figure 4: Primary siRAL sequence

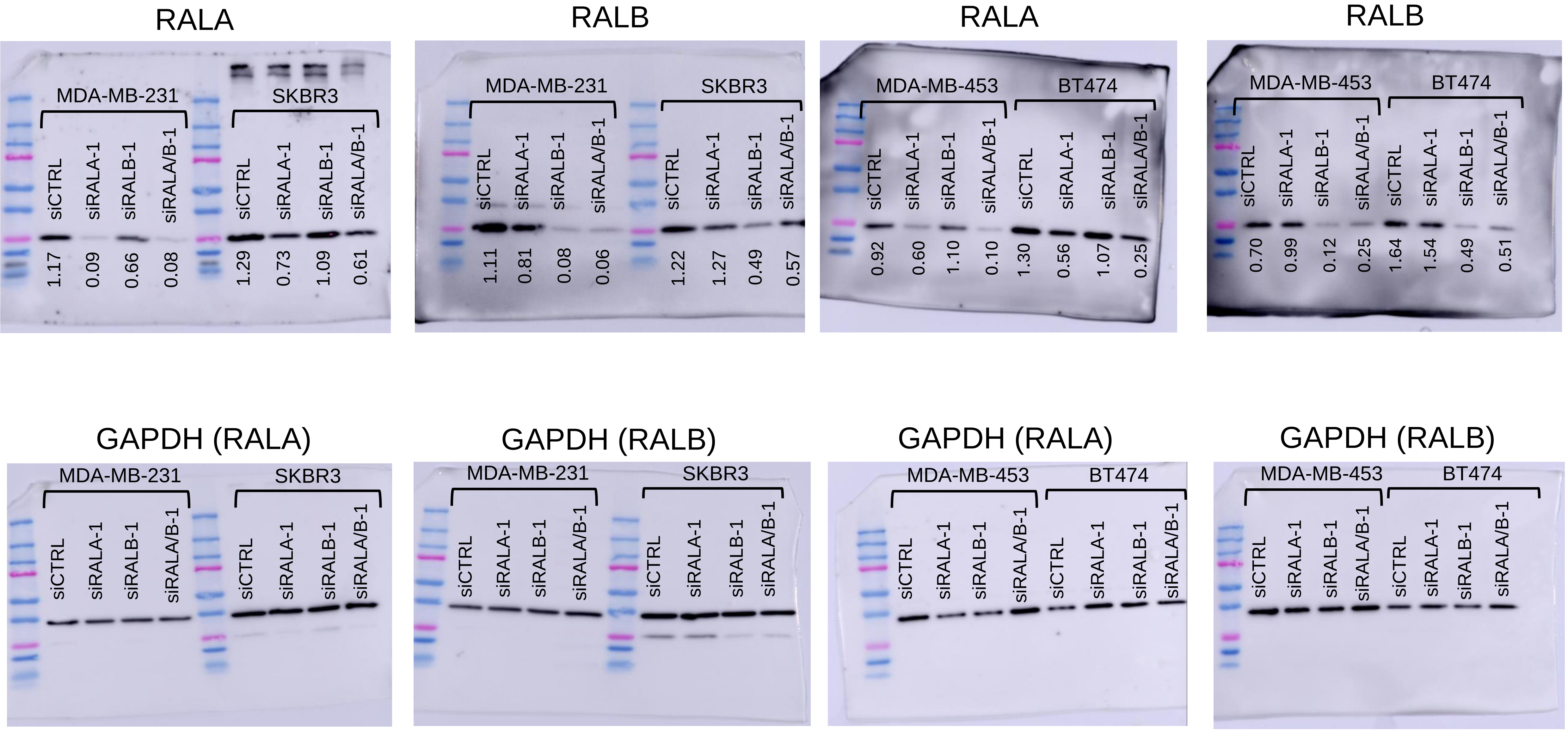

Supplementary figure 4: Primary siRAL sequence

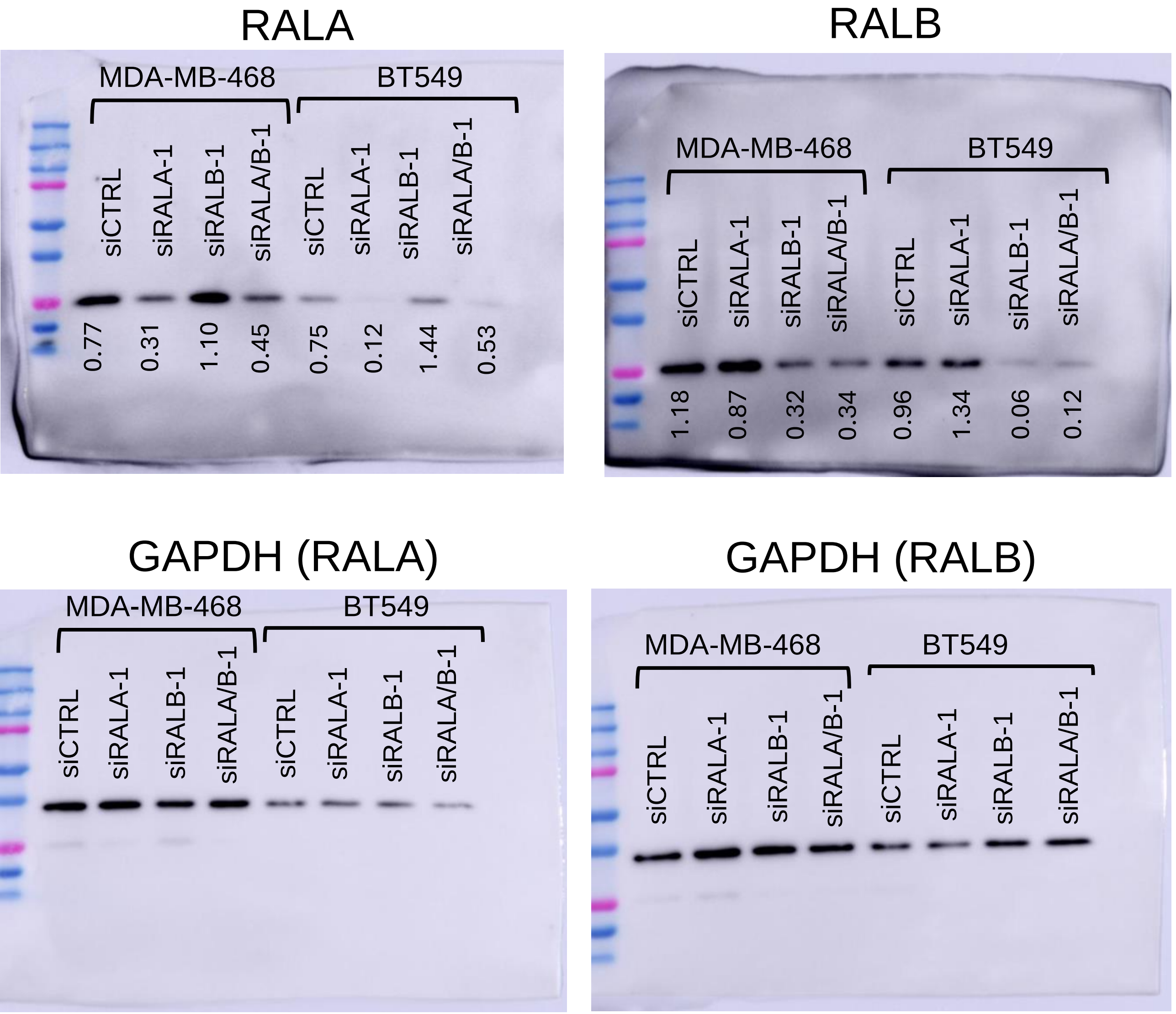

Supplementary figure 4: secondary siRAL sequence

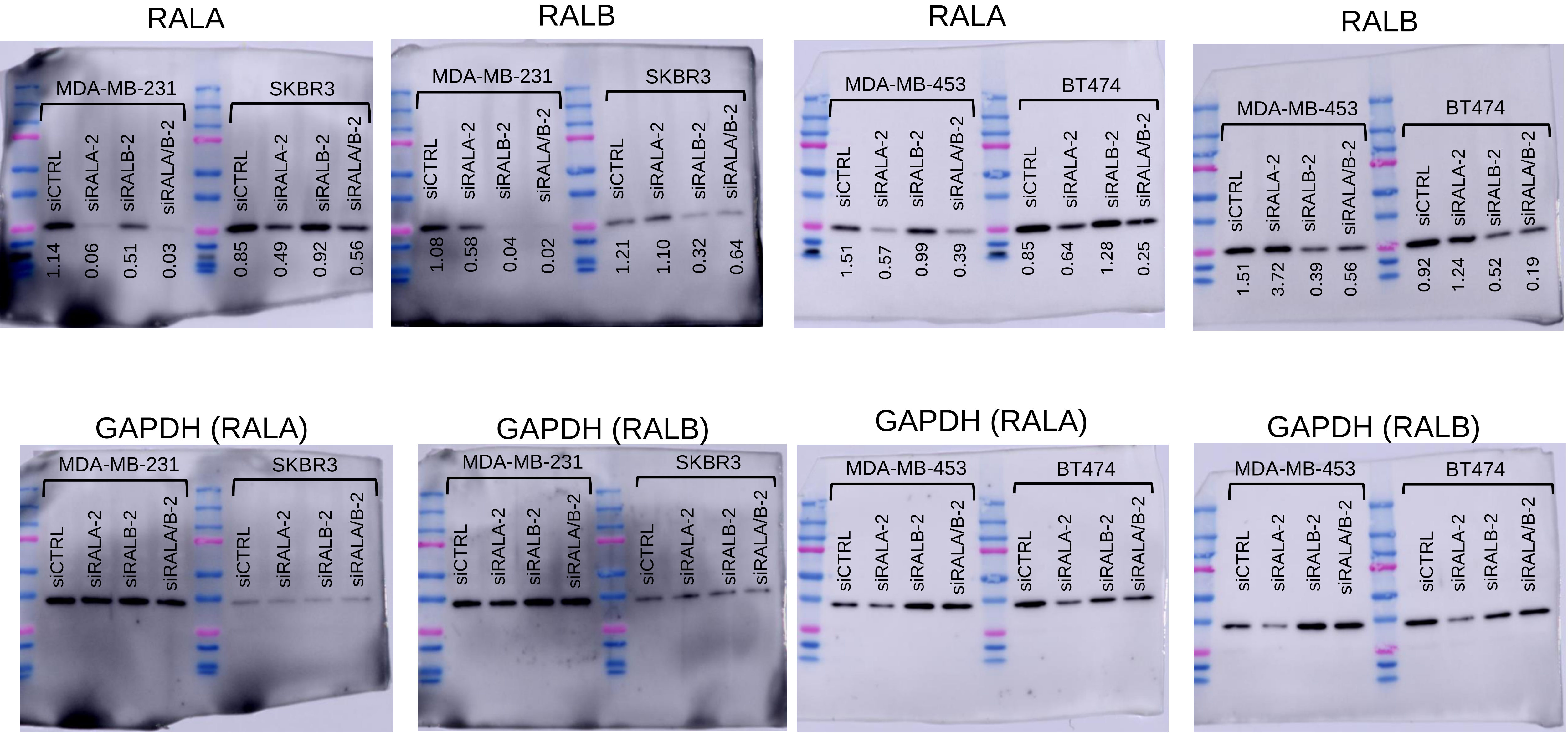

Supplementary figure 4: secondary siRAL sequence

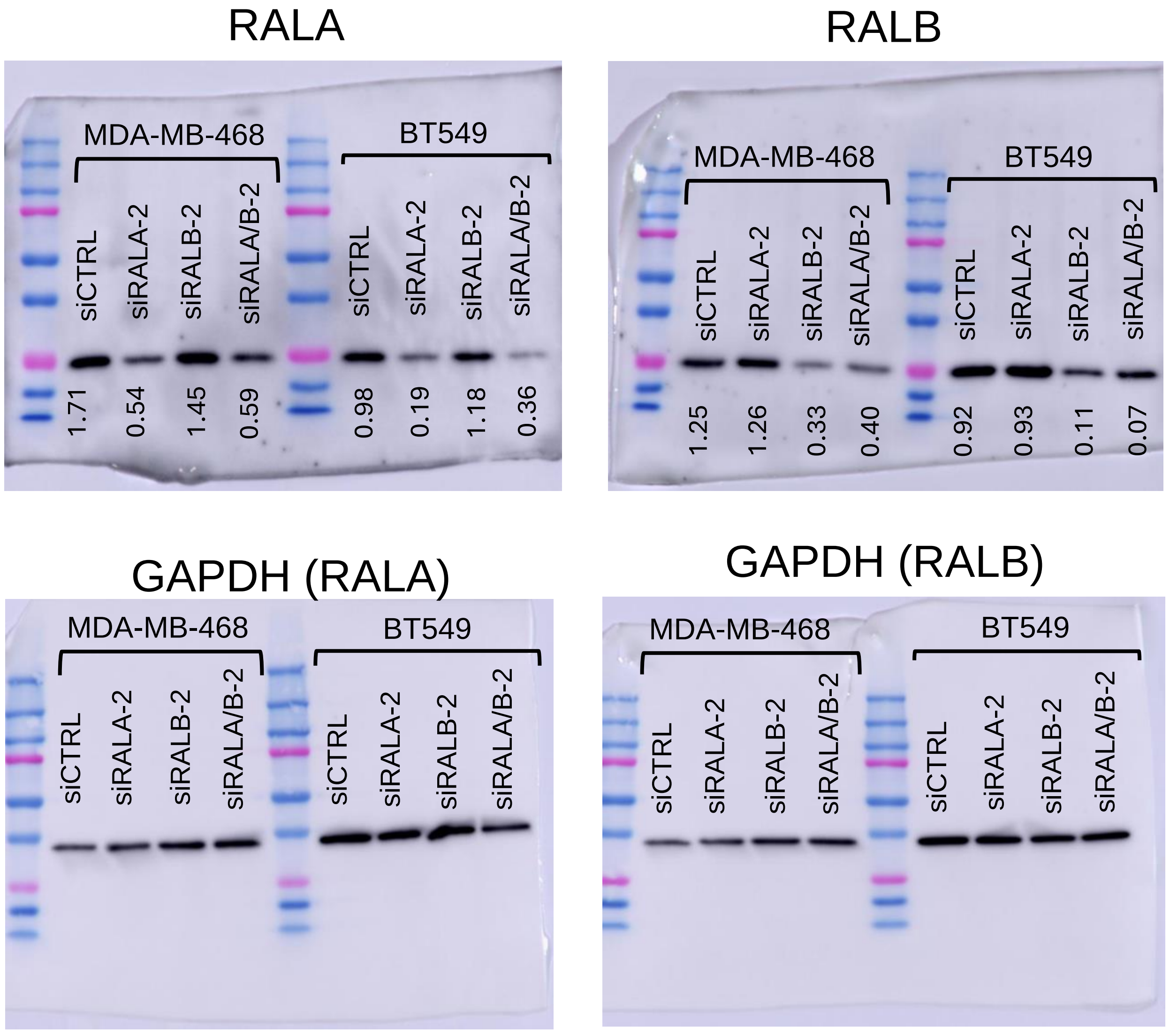

Supplementary figure 4: tertiary siRAL sequence

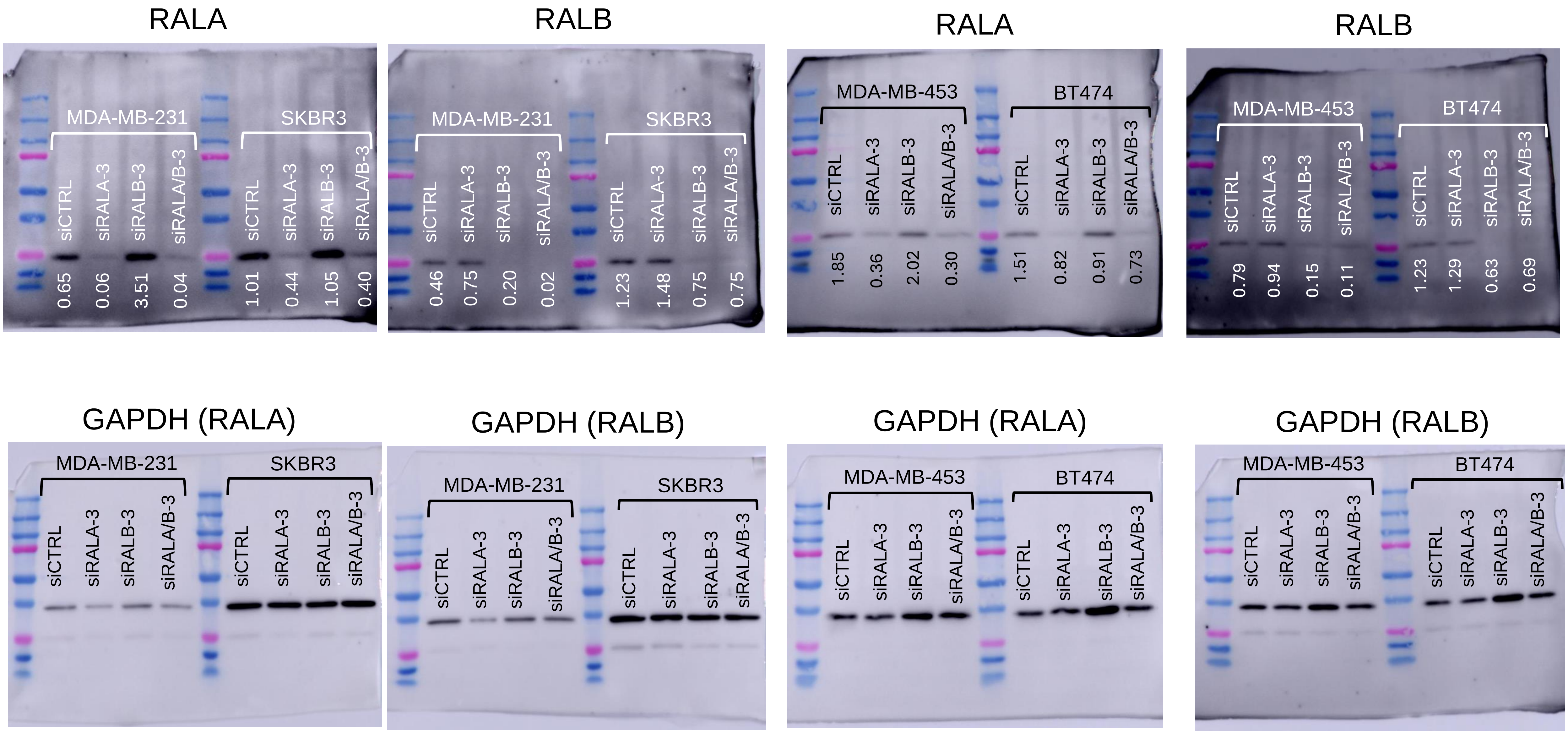

Supplementary figure 4: tertiary siRAL sequence

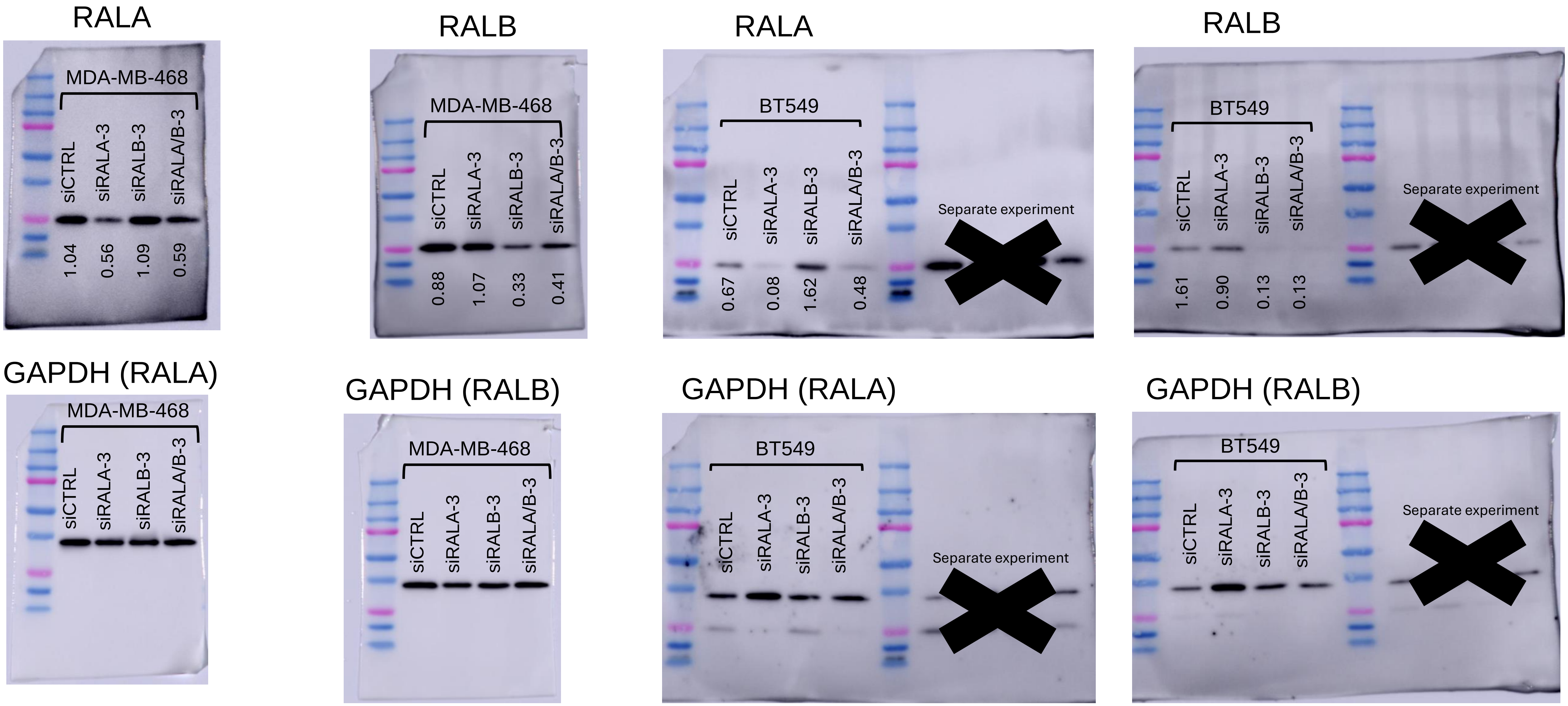

# Supplementary Figure 9A: Replicate 1

## Pan-GTP-RAS

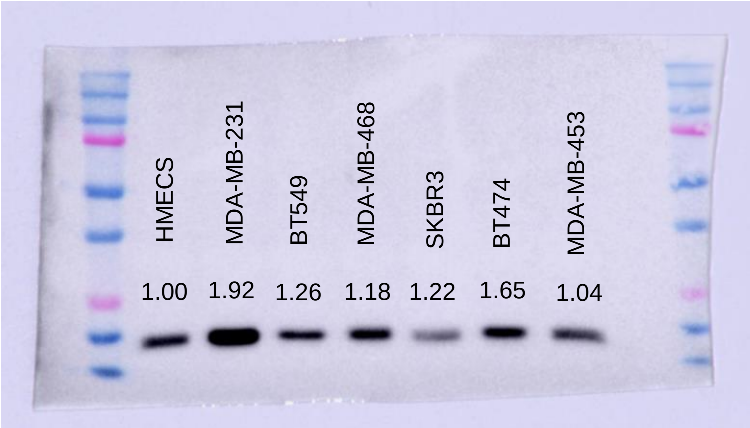


## Pan-RAS

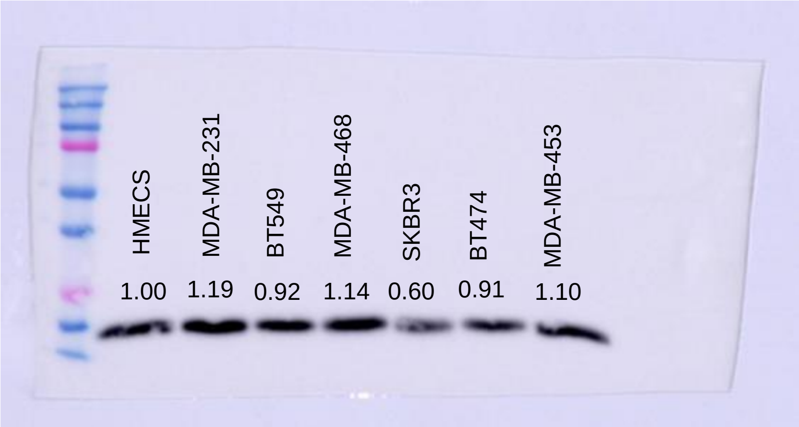


## HER2

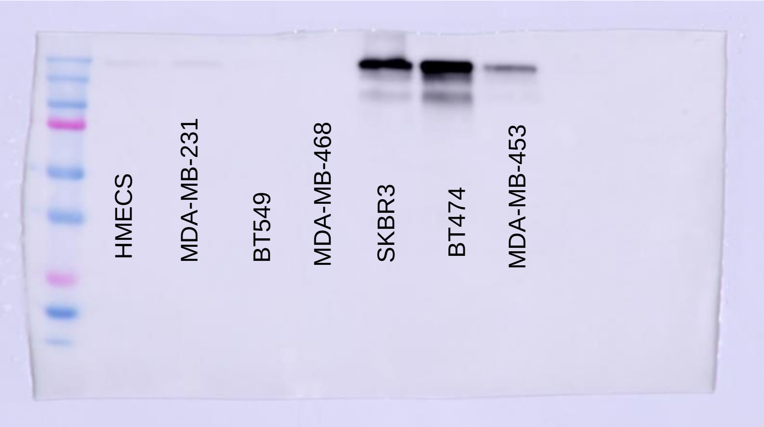


## EGFR

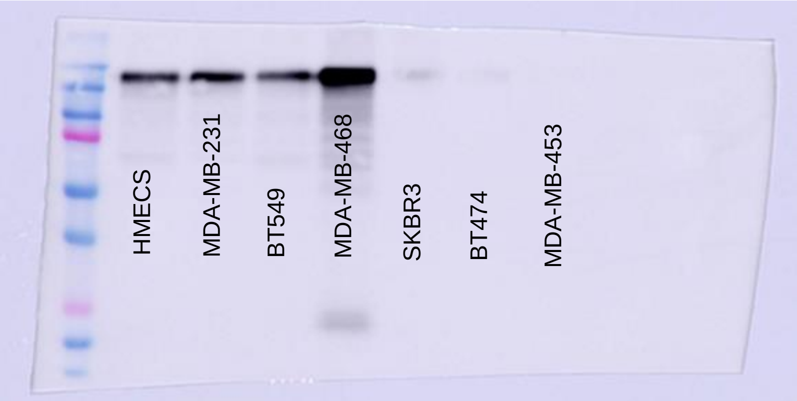


## RALB

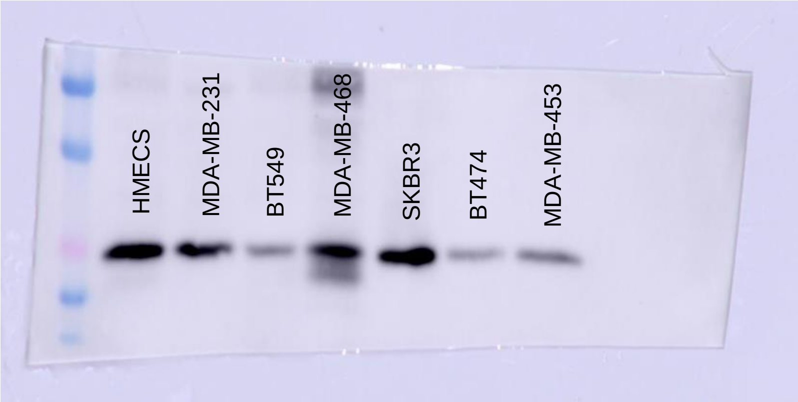


## RALA (HER2 Blot)

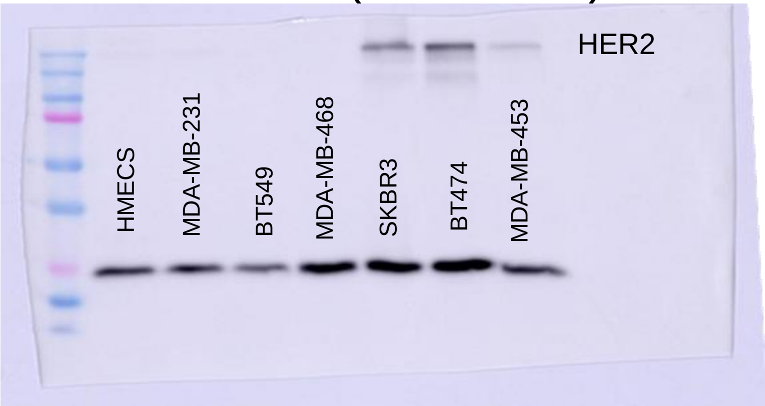


## ACTIN (RALB/EGFR )

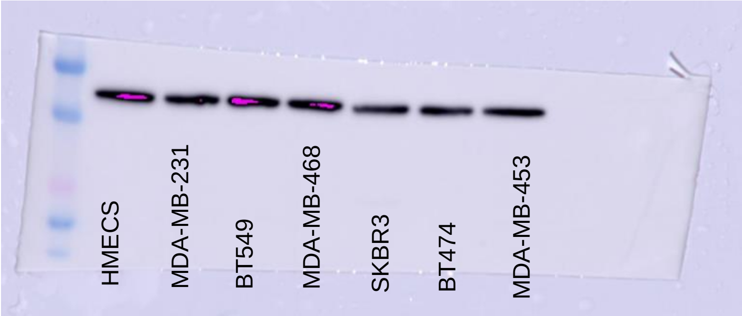


## ACTIN (Pan-RAS)

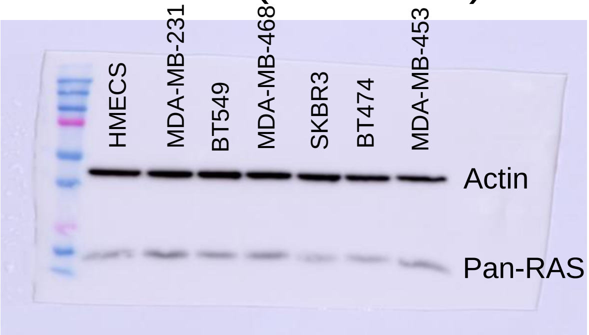


## ACTIN (HER2/RALA )

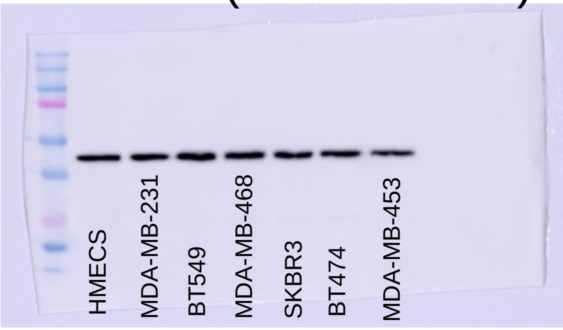

# Supplementary Figure 9A: Replicate 2

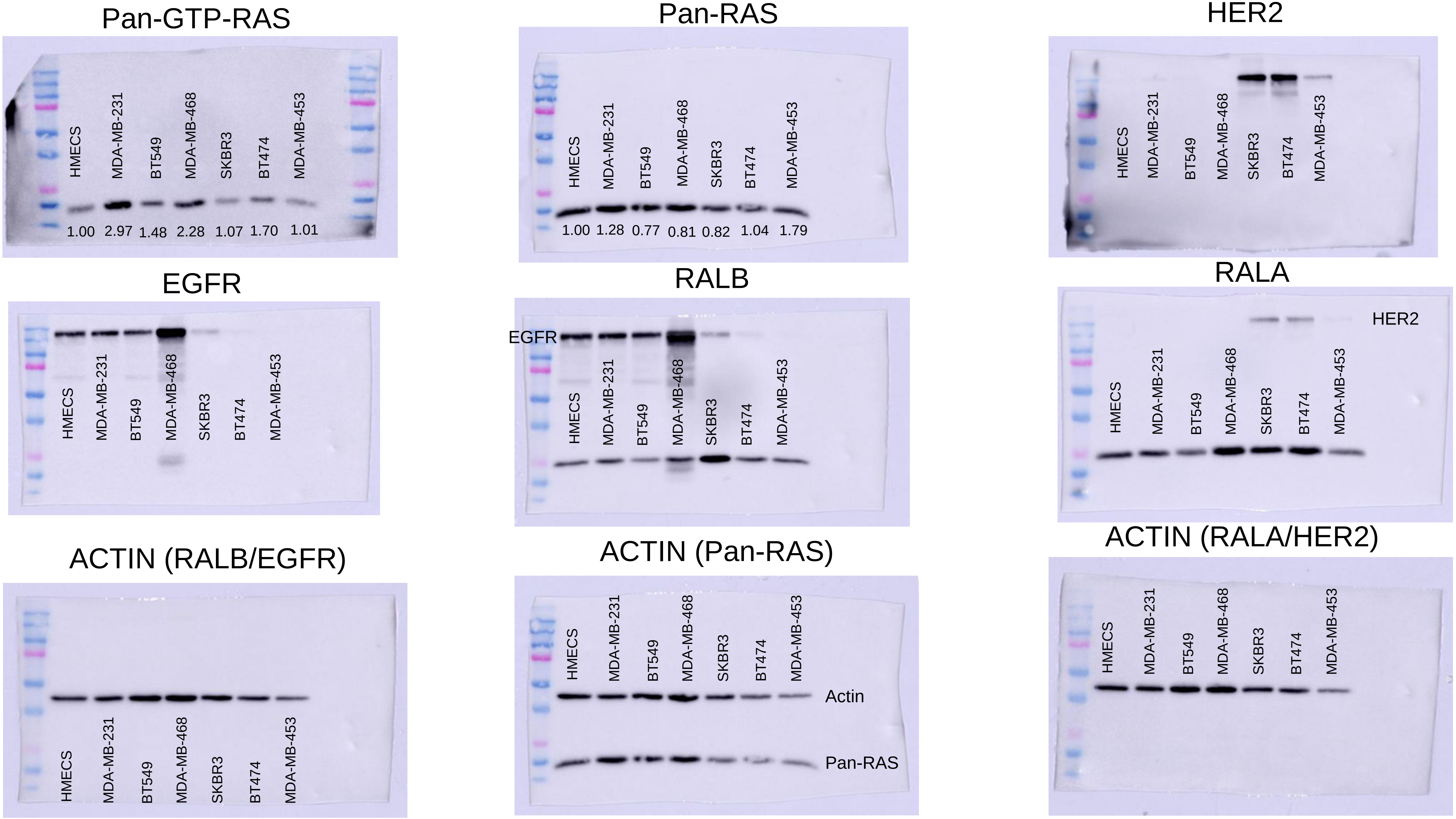

# Supplementary Figure 9E

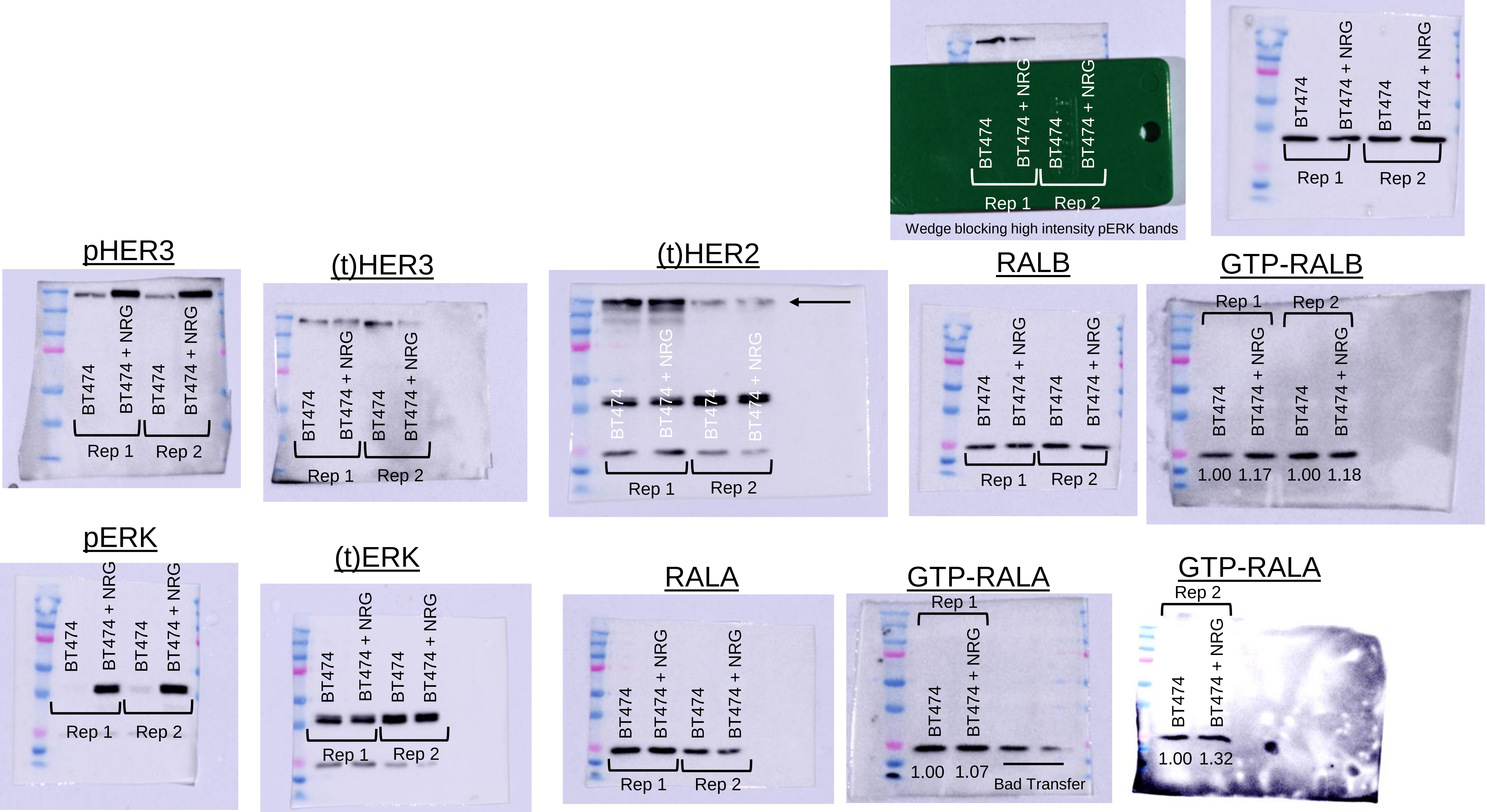

Supplementary Figure 13

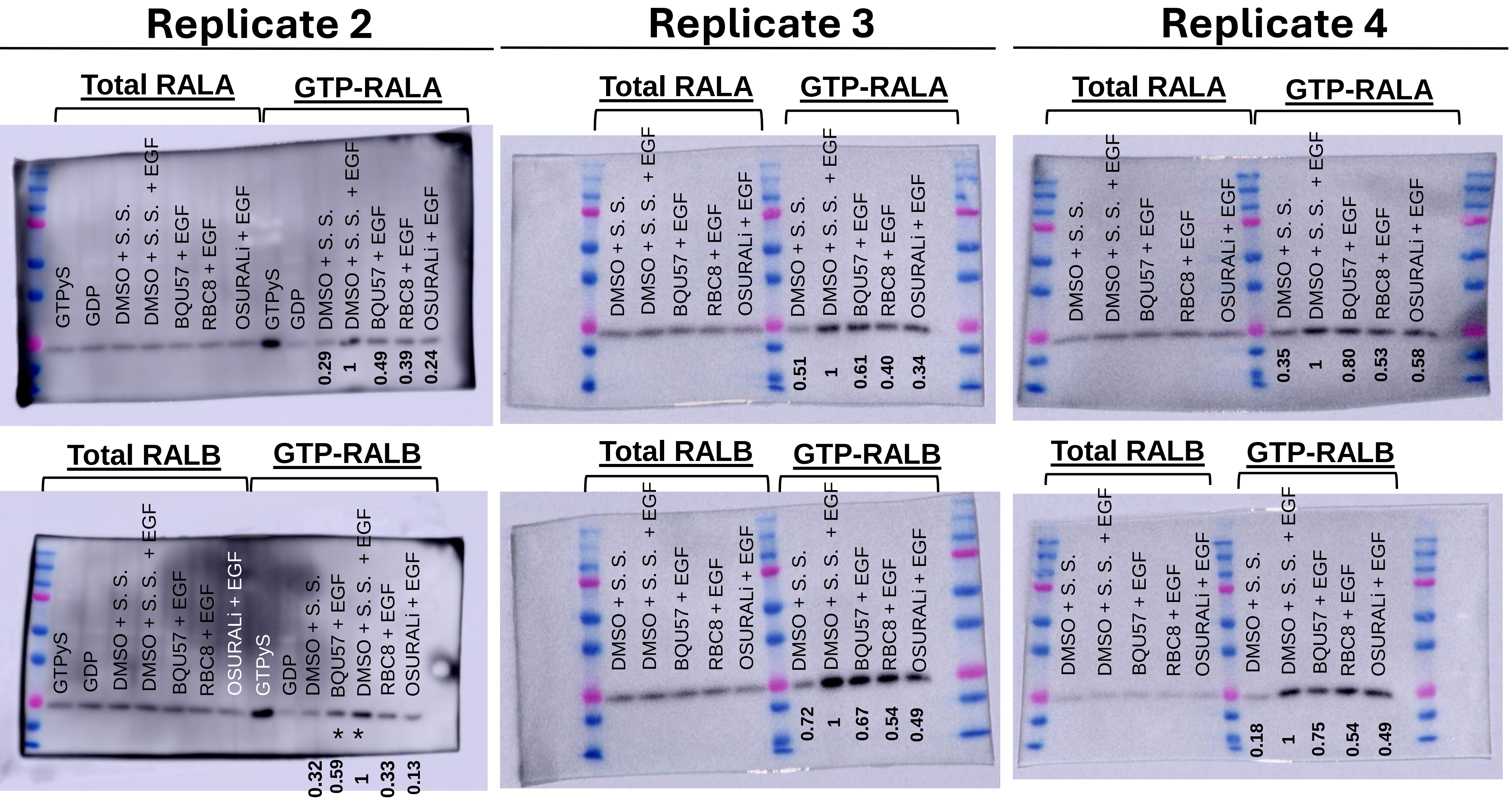

Supplement: Supplementary file 1 [file cancers-16-03043-s001.zip › File S1.pdf]
