# Supplementary material for: The RAL Small G Proteins Are Clinically Relevant Targets in Triple Negative Breast Cancer
Source: Cancers (Basel). 2024 Aug 31;16(17):3043. doi: 10.3390/cancers16173043 (PMC11394424; doi:10.3390/cancers16173043)
Supplement: Supplementary file 1 [file cancers-16-03043-s001.zip › Supplementary Tables.pdf]

S1

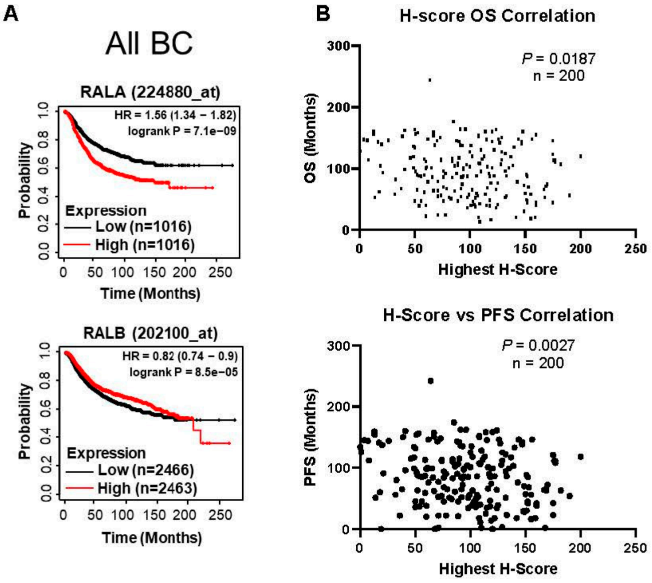

Supplemental Figure 1. (A) Kaplan-Meier analysis of BC patients including all subtypes. Grouping was accomplished by segregation between high (upper 50th percentile) vs. low expression (lower 50th percentile) groups for RALA (upper panel) and RALB (lower panel) expression with relapse-free survival as the measured outcome. Data are from KM Plotter, P<0.05. **(B)** H-score correlation data associated with our Kaplan-Meier analysis of HER2+ BC patient survival as segregated by RALA H-score based on patient sample immunostaining.

S2

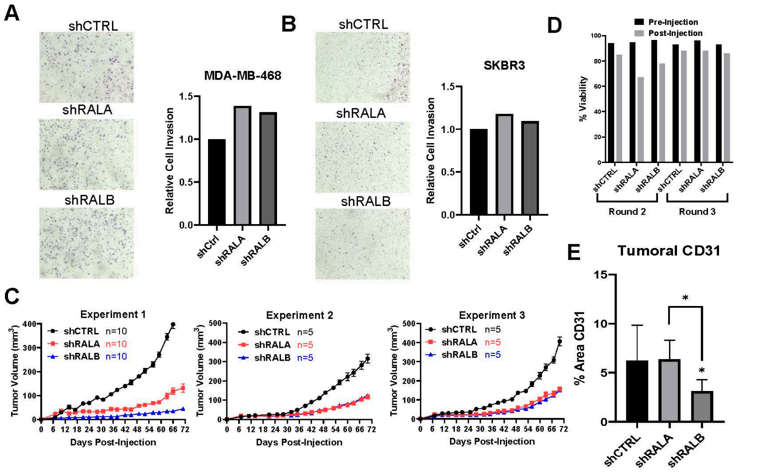

Supplemental Figure 2. Invasion assay quantification and representative images for **(A)** MDA-MD-468 (n=1) and **(B)** SKBR3 (n=1) shCtrl, shRALA, and shRALB cell lines after 24 h incubation. **(C)** MDA-MB-468 in vivo tumor growth in NSG mice was tested using three independent experiments. For Experiment 1, 10 mice were included for each group while 5 mice per group were used in Experiments 2 and 3. **(D)** Viability of the tumor cells used in Experiments 2 and 3 was determined before and after inoculation by trypan blue exclusion. **(E)** MDA-MB-468 shCTRL (n=9), shRALA (n=10), and shRALB (n=10) tumors were stained for CD31 and ROIs were made on each image separating tumor from stroma before color deconvolution to extract DAB staining. A signal threshold was then applied to the samples before measurement of the ROIs was performed to measure % area

of target staining in each region. Three representative photos from each sample were separately analyzed and mean values were used for comparisons among groups. *, P<0.05.

S3

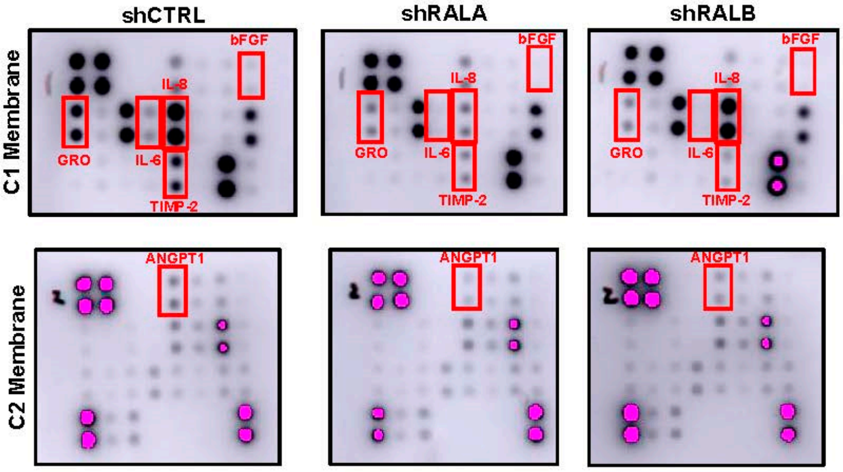

Supplemental Figure 3. Human Angiogenesis Antibody Array blots from MDA-MB-468 shCTRL, shRALA and shRALB conditioned media.

S4

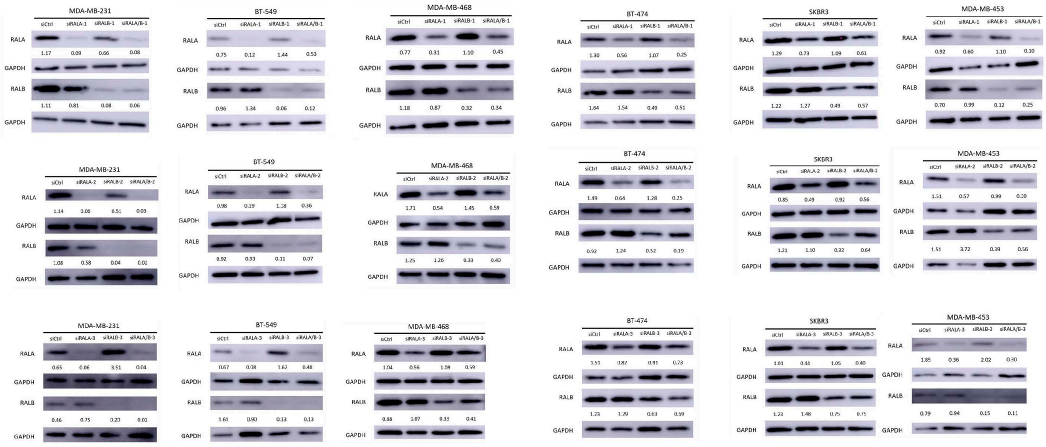

Supplemental Figure 4. Western blots displaying the relative expression of RALA and RALB in a panel of TNBC, luminal B BC, and HER2+ BC lines following transfection with the indicated siRNA.

S5

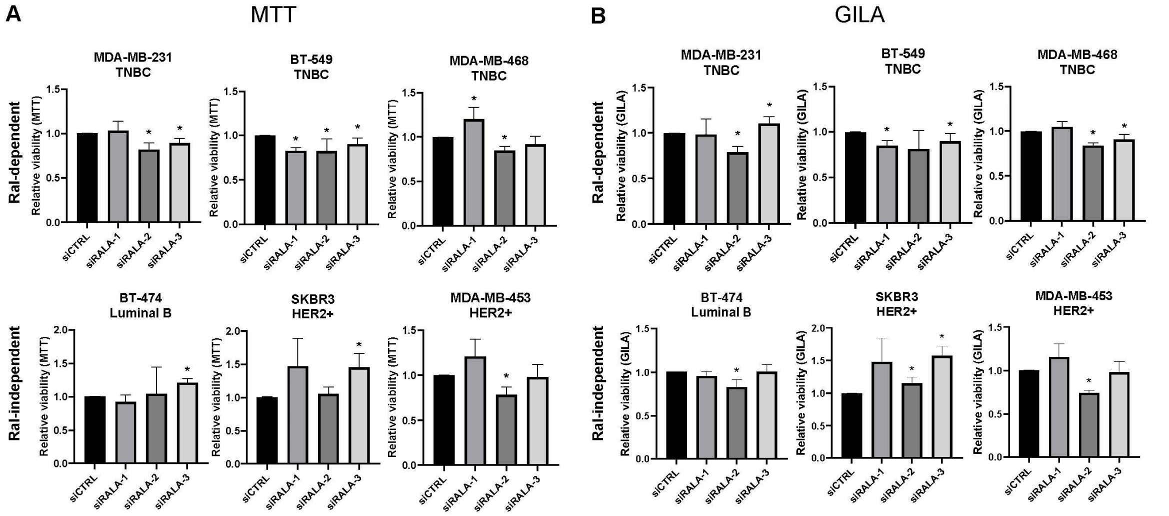

Supplemental Figure 5. (A) Quantification of cell viability over as measured by MTT. Cells were treated with the indicated siRNA sequences targeting RALA for 2 days prior to plating. Viability was measured after an additional 3 days. n=3-12. *, P<0.05. **(B)** Quantification of cell viability. Cells were treated with the indicated siRNA sequences targeting RALA for 2 days prior to plating. Viability was measured after an additional 5 days. n=3-12. *, P<0.05.

S6

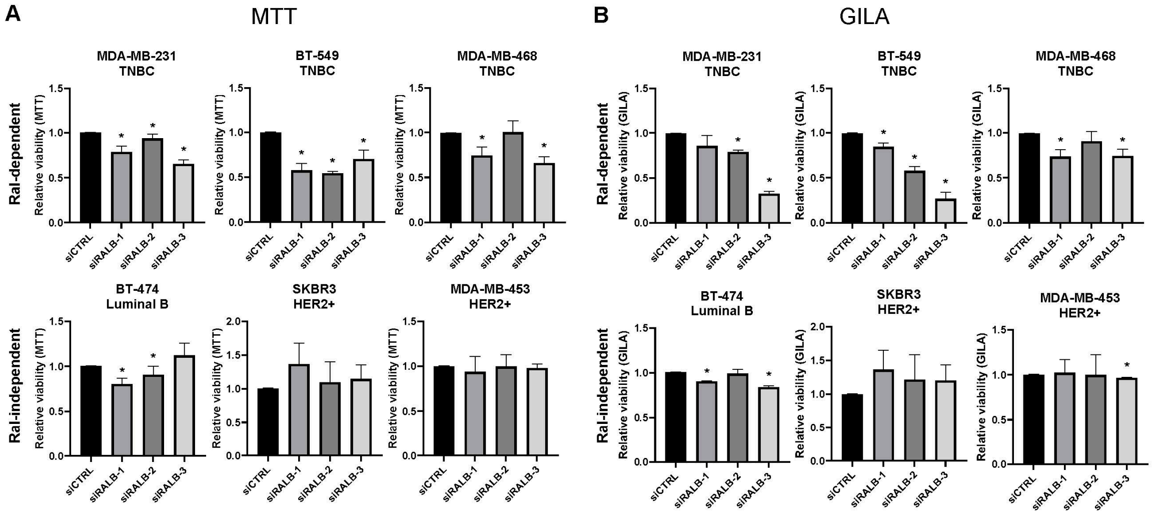

Supplemental Figure 6. (A) Quantification of cell viability over as measured by MTT. Cells were treated with the indicated siRNA sequences targeting RALB for 2 days prior to plating. Viability was measured after an additional 3 days. n=3-12. *, P<0.05. **(B)** Quantification of cell viability. Cells were treated with the indicated siRNA sequences targeting RALB for 2 days prior to plating. Viability was measured after an additional 5 days. n=3-12. *, P<0.05.

S7

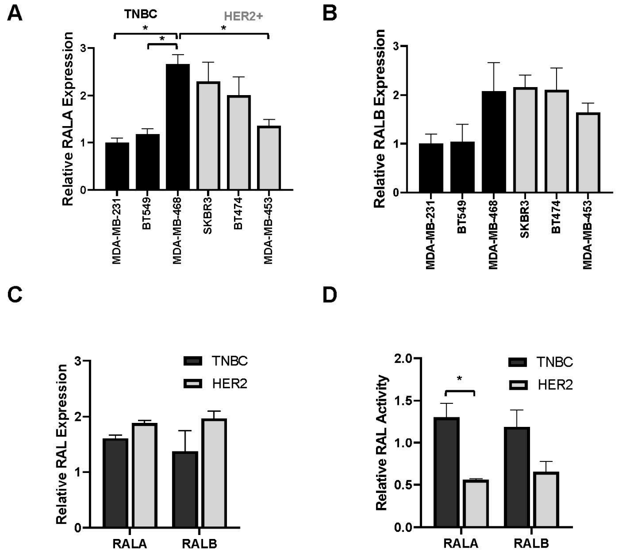

Supplemental Figure 7. (A and B) Quantification of RALA **(A)** and RALB **(B)** expression. ImageJ was used quantify RALA or RALB expression in each cell line relative to GAPDH expression. Results are presented relative to the MDA-MB-231 value (n=2). **(C and D)** Quantification of RALA (**C**) and RALB (**D**) activity. Image J was used to quantify GTP-bound RALA or RALB relative to total RALA or RALB in each cell line. Results are presented relative to the MDA-MB-231 value (n=2). *, P<0.05.

S8

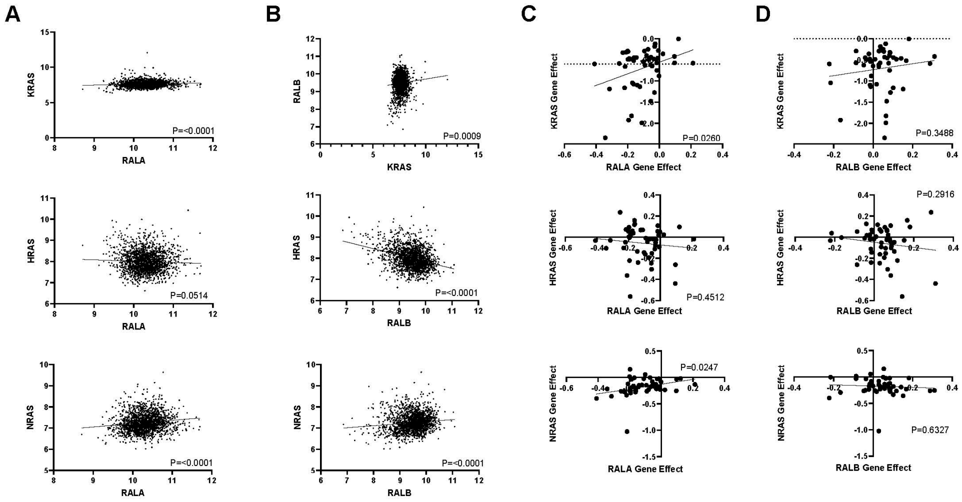

Supplemental Figure 8. (A) Pearson correlation of *RALA* gene expression with gene expression of *KRAS*, *HRAS*, or *NRAS* in the METABRIC breast cancer patient cohort. **(B)** Pearson correlation of *RALB* gene expression with gene expression of *KRAS*, *HRAS*, or *NRAS* in the METABRIC breast cancer patient cohort. Data from CRISPR loss-of-function screens performed on 48 human breast cancer cell lines in the Cancer Dependency Map Project (DepMap) was used to determine Pearson correlation of cell line dependency on RALA **(C)** or RALB **(D)** with cell line dependency on members of the RAS family.

S9

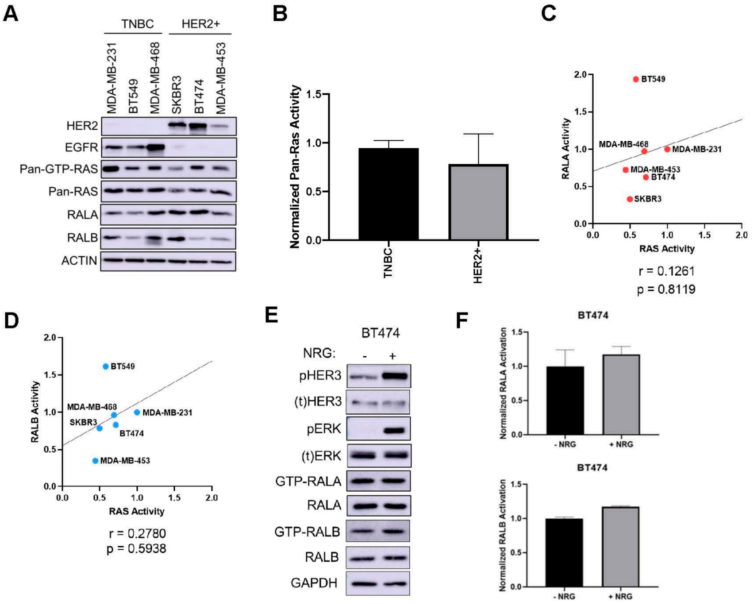

Supplemental Figure 9. (A) Representative western blots illustrating HER2 and EGFR expression in selected TNBC and HER2+ breast cancer cell lines as well as active GTP-bound pan-RAS and total pan-RAS as determined by RAS pulldown activity assay. **(B)** Bar graphs summarize the relative RAS activity in TNBC (MDA-MB-231, BT549, MDA-MD-468) and HER2+ (SKBR3, BT474, MDA-MB-453) cell lines. RAS activity in each cell line was determined in two independent experiments. **(C and D)** Plots illustrate the correlation between RAS activity and RALA **(C)** or RALB **(D)** activity in the indicated cell lines. RAS and RAL activity were determined by pulldown assay. Each dot represents the average of at least two activity assays. Correlation was determined by Pearson's method. **(E)** BT474 cells were stimulated by neuregulin (NRG) and cell lysates were collected for western blot and RAL activity pulldown assay. Representative western blots are shown. **(F)** Bar graphs summarize RALA (upper) and RALB (lower) in BT474 cells stimulated by NRG. Results are the average of two independent experiments.

S10

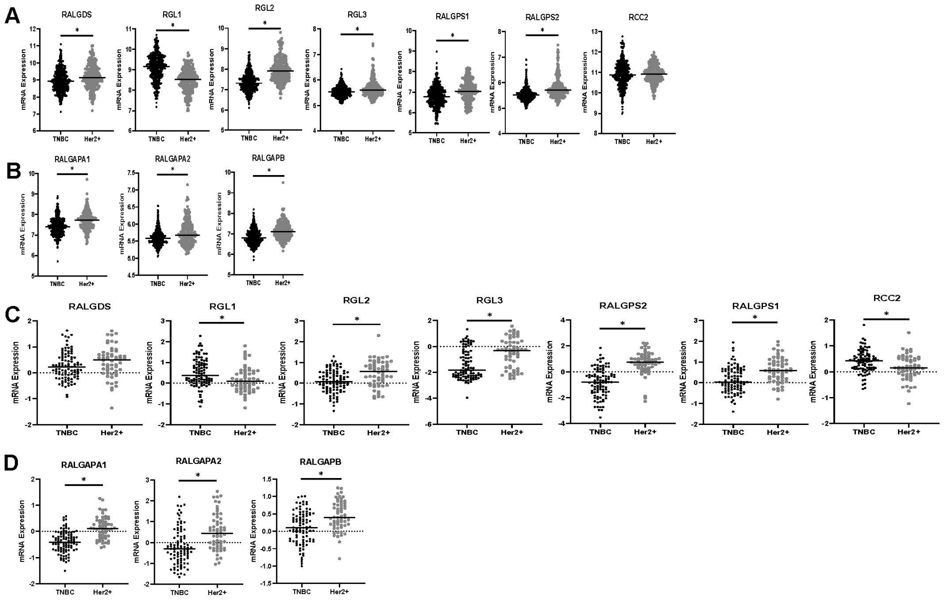

Supplemental Figure 10. Expression of RALGEFs **(A)** and RALGAPs **(B)** expression in TNBC (n = 398) and HER2+ (n = 220) patients in the METABRIC breast cancer patient cohort. Expression of RALGEFs **(C)** and RALGAPs **(D)** in TNBC (n = 95) and HER2+ (n = 58) patients in the TCGA BRCA breast cancer patient cohort. *, P<0.05.

S11

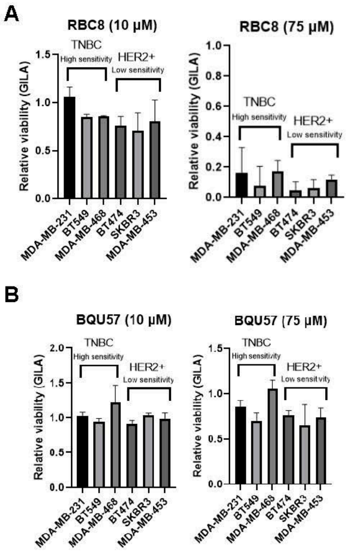

Supplemental Figure 11. (A) Viability data from figure 5A displayed at selected RBC8 concentrations to enable comparison by subtype. **(B)** Viability data from figure 5A displayed at selected BQU57 concentrations to enable comparison by subtype.

S12

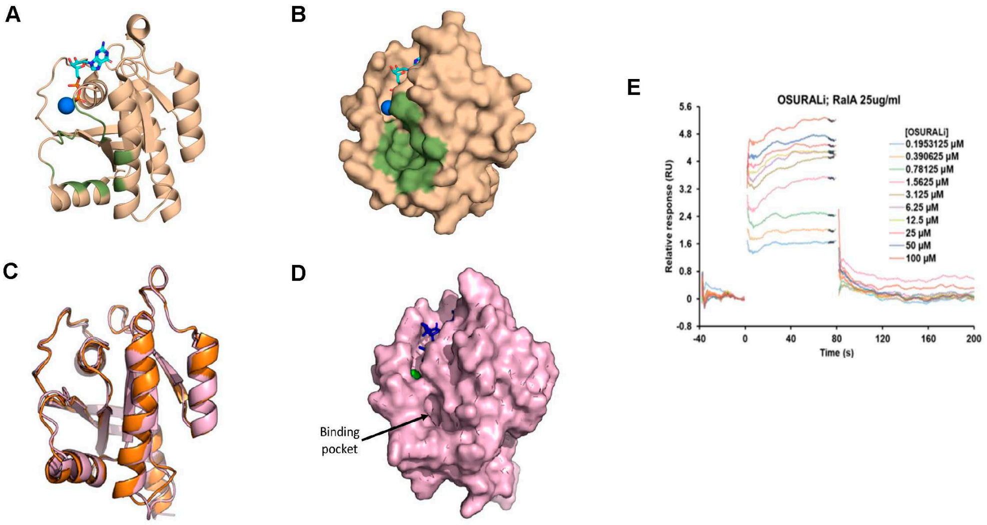

Supplemental Figure 12. Cartoon representation **(A)** and surface view **(B)** of the RALA-GDP (2BOV) crystal structure. GDP is shown in light blue and the Mg^{2+} is shown in dark blue. The residues forming the targeted allosteric binding site are shown in green. Cartoon representation **(C)** and surface representation **(D)** of the homology model of RALB-GDP with the lowest Rosetta score (S_15_0062). GDP is shown in blue and the Mg^{2+} is shown in green. The targeted allosteric binding site it marked by an arrow. RalA-GDP crystal structure is shown in orange for comparison. **(E)** Sensorgrams of OSURALi interactions with recombinant RALA obtained via SPR.

S13

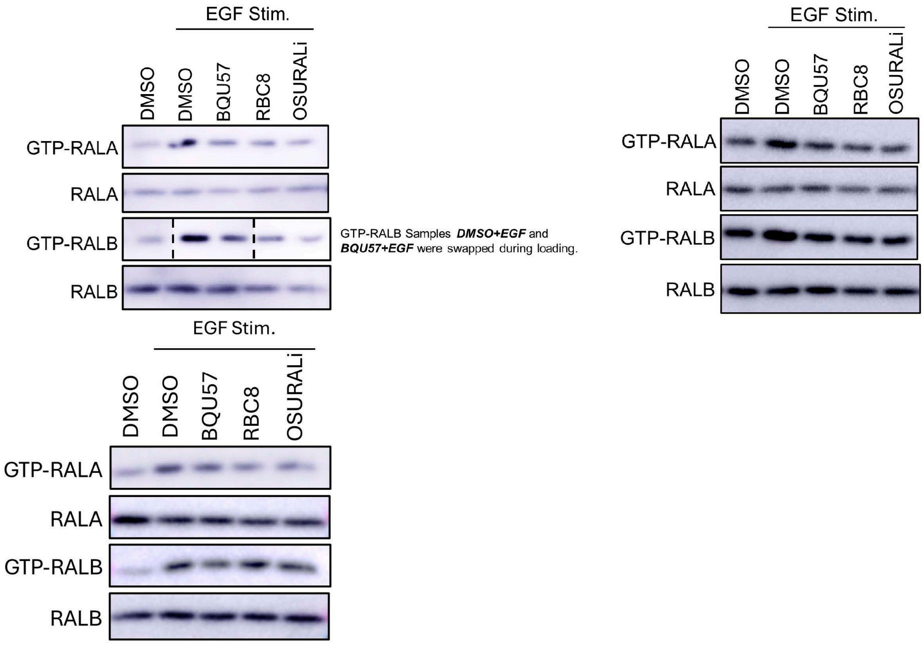

Supplemental Figure 13. Individual replicates of western blots illustrating inhibition of RALA and RALB activity by BQU57, RBC8, and OSURALi. MDA-MB-468 cells were pre-incubated with 50uM of RAL inhibitor for 1h prior to stimulation of RAL activity by EGF.
